# [Corrigendum] Morin exerts cytoprotective effects against oxidative stress in C2C12 myoblasts via the upregulation of Nrf2-dependent HO-1 expression and the activation of the ERK pathway

**DOI:** 10.3892/ijmm.2025.5555

**Published:** 2025-05-28

**Authors:** Moon Hee Lee, Min Ho Han, Dae-Sung Lee, Cheol Park, Su-Hyun Hong, Gi-Young Kim, Sang Hoon Hong, Kyoung Seob Song, Il-Whan Choi, Hee-Jae Cha, Yung Hyun Choi

Int J Mol Med 39: 399-406, 2017; DOI: 10.3892/ijmm.2016.2837

Following the publication of the above article, an interested reader drew to the authors' attention that the β-actin blots in Figs. 4B and 5B were apparently the same, even though different experimental conditions were reported for these figure parts; moreover, the data shown as the Lamin B blot in Fig. 5A had also been incorporated as the Akt data in Fig. 5C of a different paper submitted by the same group and published in the journal *Oncology Reports*.

However, the authors were able to re-examine their data, and realized how these errors had occurred. Upon repeating the experiments relating to the affected figure parts, which resulted in the generation of similar results, revised versions of Figs. 4 and 5, now including replacement data for Fig. 4A and Fig. 5A and B, are shown on the next page. Note that the errors made with the assembly of the data in these figures did not affect the overall conclusions reported in the paper. The authors are grateful to have been given the opportunity to publish this corrigendum, and all the authors agree with its publication; furthermore, they apologize to the Editor of *International Journal of Molecular Medicine* and to the readership for any inconvenience caused.

## Figures and Tables

**Figure 4:**
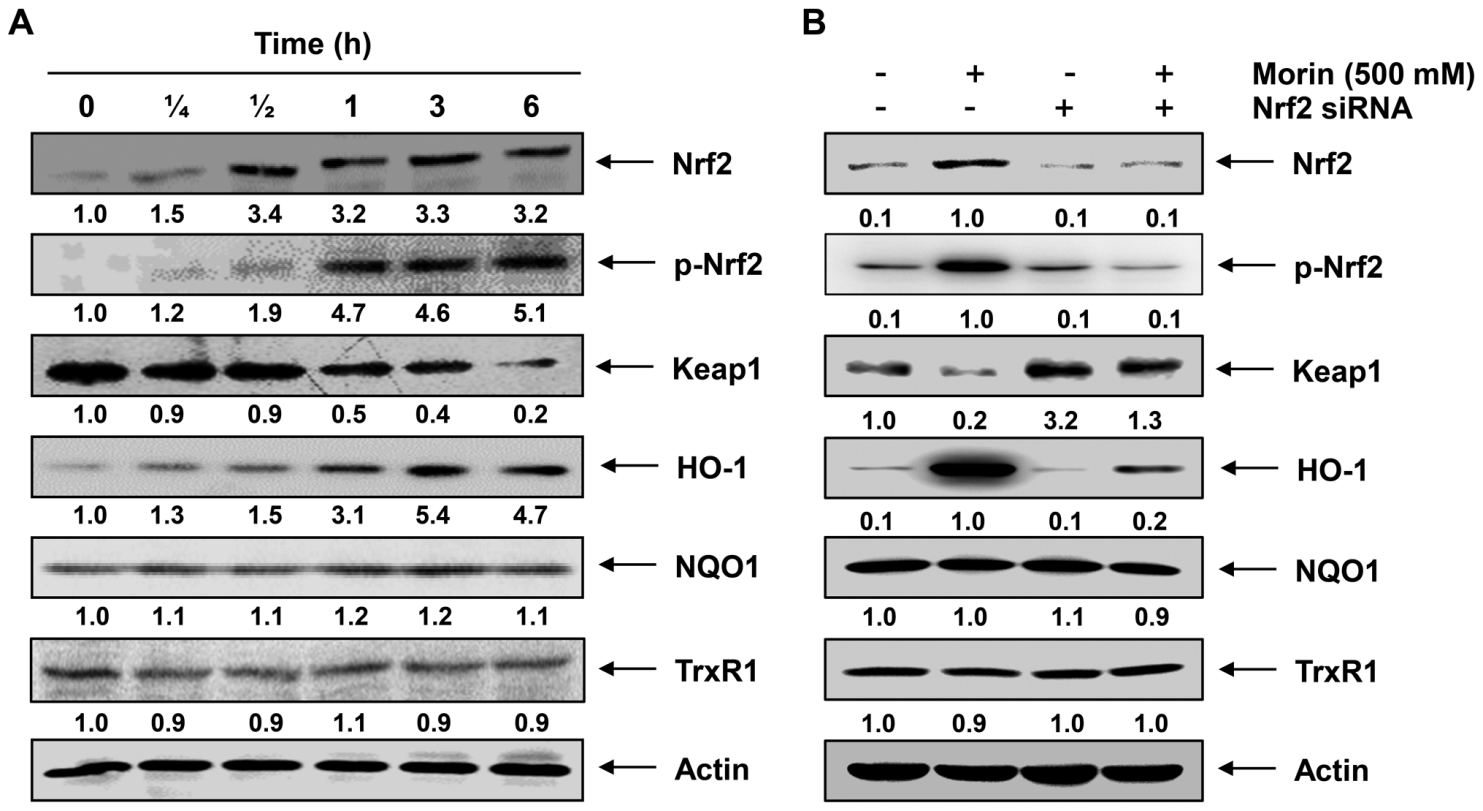
Morin induces nuclear factor-erythroid 2-related factor 2 (Nrf2)-dependent heme oxygenase-1 (HO-1) expression in C2C12 myoblasts. (A) Cells were incubated with 500 μM morin for the indicated time periods. The cells were lysed and then equal amounts of cell lysates were separated on sodium dodecyl sulfate (SDS)-polyacrylamide gels and transferred to nitrocellulose membranes. (B) Cells were transfected with Nrf2 siRNA. After 24 h, the cells were treated with or without 500 μM morin for 6 h. The proteins were separated on SDS-polyacrylamide gels and then transferred onto nitrocellulose membranes. The membranes were probed with the specific antibodies against Nrf2, p-Nrf2, Keap1 and HO-1. Proteins were visualized using an enhanced chemiluminescence (ECL) detection system. Actin was used as an internal control. The relative ratios of expression in the western blotting results are shown at the bottom of each of the results as relative values to actin expression.

**Figure 5:**
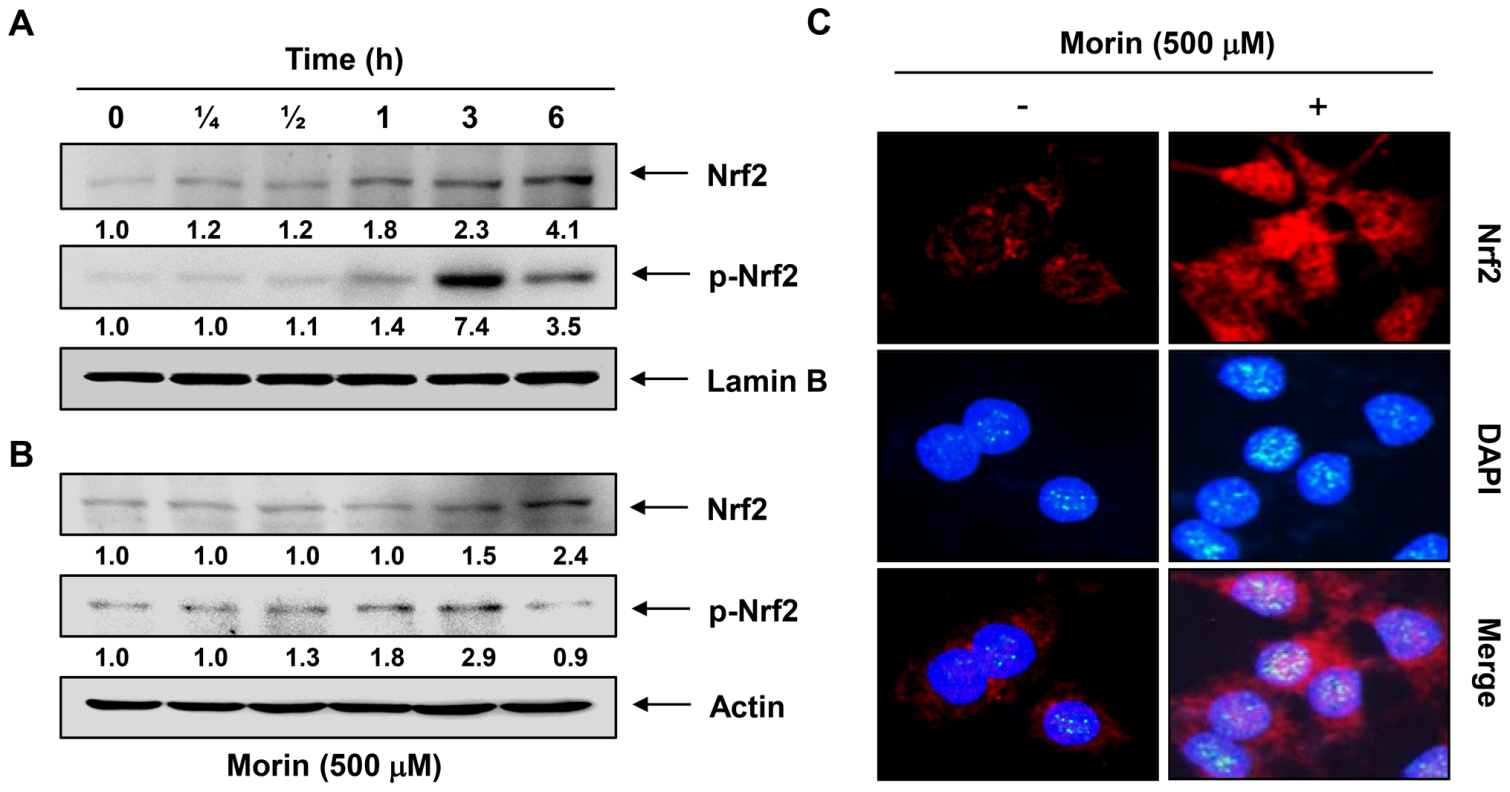
Morin enhances nuclear translocation of nuclear factor-erythroid 2-related factor 2 (Nrf2) in C2C12 myoblasts. (A and B) Cells were incubated with 500 μM morin for the indicated time periods. (A) Nuclear or (B) cytosolic proteins were separated on sodium dodecyl sulfate (SDS)-polyacrylamide gels and then transferred onto nitrocellulose membranes. The membranes were probed with anti-Nrf2 and anti-p-Nrf2 antibodies. Proteins were visualized using an enhanced chemiluminescence (ECL) detection system. Lamin B and actin were used as the internal controls of nuclear and cytosolic proteins, respectively. The relative ratios of expression in the western blotting results are shown at the bottom of each of the results as relative values to lamin B (A) or actin (B) expression. (C) Cells were treated with 500 μM morin for 6 h and then localization of Nrf2 was visualized with a fluorescence microscope after immunofluorescence staining with anti-Nrf2 antibody and an FITC-labeled anti-rabbit IgG antibody (red). Nuclei of the corresponding cells were visualized with DAPI (blue).

